# Risk factors of delayed recovery from general anesthesia in patients undergoing radical biliary surgery: What can we prevent

**DOI:** 10.1097/MD.0000000000026773

**Published:** 2021-08-13

**Authors:** Guohui Zhang, Bingbing Pan, Dan Tan, Yingzi Ling

**Affiliations:** Department of Anesthesia (Clinical Research Center for Anesthesiology of ERAS in Hunan Province), Hunan Provincial People's Hospital (First Affiliated Hospital of Hunan Normal University), Changsha, P.R. China.

**Keywords:** biliary surgery, delayed recovery, general anesthesia, laparoscopic, nursing, treatment

## Abstract

Delayed recovery (DR) is very commonly seen in the patients undergoing laparoscopic radical biliary surgery, we aimed to investigate the potential risk factors of DR in the patients undergoing radical biliary surgery, to provide evidences into the management of DR.

Patients who underwent radical biliary surgery from January 1, 2018 to August 31, 2020 were identified. The clinical characteristics and treatment details of DR and no-DR patients were compared and analyzed. Multivariable logistic regression analyses were conducted to identify the potential influencing factors for DR in patients with laparoscopic radical biliary surgery.

We included a total of 168 patients with laparoscopic radical biliary surgery, the incidence of postoperative DR was 25%. There were significant differences on the duration of surgery, duration of anesthesia, and use of intraoperative combined sevoflurane inhalation (all *P* < .05), and there were not significant differences on American Society of Anesthesiologists, New York Heart Association, tumor-lymph node- metastasis, and estimated blood loss between DR group and control group (all *P* > .05). Multivariable logistic regression analyses indicated that age ≥70 years (odd ratio [OR] 1.454, 95% confidence interval [CI] 1.146–1.904), body mass index ≥25 kg/m^2^ (OR 1.303, 95% CI 1.102–1.912), alcohol drinking (OR 2.041, 95% CI 1.336–3.085), smoking (OR 1.128, 95% CI 1.007–2.261), duration of surgery ≥220 minutes (OR 1.239, 95% CI 1.039–1.735), duration of anesthesia ≥230 minutes (OR 1.223, 95% CI 1.013–1.926), intraoperative combined sevoflurane inhalation (OR 1.207, 95% CI 1.008–1.764) were the independent risk factors for DR in patients with radical biliary surgery (all *P* < .05).

It is clinically necessary to take early countermeasures against various risk factors to reduce the occurrence of DR, and to improve the prognosis of patients.

## 1 Introduction

Hilar cholangiocarcinoma accounts for 50% to 70% of all cholangiocarcinomas.^[1]^ The anatomical location of the tumor is special, the surrounding structure is complicated, and because of its invasive growth characteristics, it easily invades the hepatic artery and portal vein.^[2]^ It has been reported that if treated with early radical resection, the 1-year survival rate of patients is 80% and the 5-year survival rate is 39%.^[3,4]^ At present, surgical resection is the first choice for the treatment of hilar cholangiocarcinoma, and the choice of surgical method mainly depends on the extent of local invasion of the cancer.^[5]^ Because of its special anatomical relationship and biological characteristics, the rate of radical surgery is not high, and the prognosis of patients is poor.^[6]^ Therefore, the early diagnosis and treatment is vital to the prognosis of patients.

Delayed recovery (DR) from anesthesia generally refers to that the patient has not recovered 120 minutes after the end of general anesthesia, and cannot respond correctly to external stimuli and language commands.^[7]^ With the continuous development of anesthetics and related monitoring technologies, the incidence of delayed recovery has been declining year by year, but it is still one of the main complications of general anesthesia.^[8]^ It not only increases the perioperative risk, affects the recovery of the patient's physical function, but also increases the economic burden of the patient, and even causes death.^[9]^ Due to the long operation time and the large amount of anesthetic drugs in laparoscopic radical biliary surgery, the risk of DR after surgery also increases.^[10]^ Therefore, early detection of the risk factors for DR from anesthesia after laparoscopic radical biliary surgery is of crucial importance to reduce its incidence and ensure perioperative safety key. In recent years, most of the researches on high-risk factors for delayed recovery after general anesthesia have been limited to certain anesthetics or certain age groups, and there are few studies on DR after radical biliary tract surgery. In this present study, we analyzed the high-risk factors of DR after laparoscopic radical biliary surgery under general anesthesia, and aimed to provide a theoretical basis for the prevention of DR in patients with laparoscopic radical biliary surgery, thereby providing evidence to the management of DR.

## 2 Methods

### 2.1 Ethical consideration

This present study was approved by the medical ethical committee of our hospital (160,933). We applied a retrospective study design, we had informed the patients that their treatment data might be used for research and the written informed consents had been obtained from all the included patients.

### 2.2 Patients

Patients who underwent laparoscopic radical biliary surgery treated in our hospital from January 1, 2018 to August 31, 2020 were identified as potential patients. The inclusion criteria for patients were as follows: according to the preoperative computed tomography, magnetic resonance imaging, and color Doppler ultrasound examinations, it was clear that there was hilar space occupation, and the intraoperative or postoperative pathological report confirmed the diagnosis of hilar bile duct cancer.^[11]^ The related characteristics and clinical pathological data were complete. Patients underwent general anesthesia; patients were well informed and signed the written informed consents. The exclusion criteria were: patients with duodenum, pancreas, and ampullary diseases that might require bile duct anastomosis; those patients did not agree to participant in our study.

### 2.3 Laparoscopic radical biliary surgery

The laparoscopic radical biliary surgery was performed by the same group of surgeons. All patients underwent partial hepatectomy, extrahepatic bile duct resection, regional lymph node dissection, lymph node biopsy, and Roux-en-Y bile duct jejunostomy. The laparoscopic radical biliary surgery was conducted as previous studies^[12,13]^ reported. A biliary drainage catheter was used in all patients, and a grooved drainage tube was routinely placed near the liver section and anastomosis.

### 2.4 Data collection

We recorded and collected the patient's basic information, medical history, and auxiliary examinations and other indicators before surgery, including sex, age, body mass index (BMI), smoking history, hypertension, diabetes, hyperlipidemia, anemia. And we collected and analyzed the surgery and anesthesia related information, including American Society of Anesthesiologists score, New York Heart Association, tumor (topography) - lymph node- metastasis (TNM) classification data (TNM Level I: The cancer tissue is limited to the place where the initial formation is initially formed, and there is no sign of diffusion. Level II: Cancer cells have spread to nearby lymph nodes, tissues or organs; Level III: Cancer cells have spread to multiple organs or tissues of the body), duration of surgery, duration of anesthesia, estimated blood loss, the use of intraoperative combined sevoflurane inhalation.

After the operation, all the patients were sent to the anesthesia recovery room, and we evaluated the patient every 15 minutes according to the Steward awakening score.^[14,15]^ The Steward awakening score was divided into 3 dimension including awakeness, unobstructed breathing, extent of physical activity. Steward score <4 points was regarded as not awakened, Steward score ≥4 points was regarded as awakened. According to the Steward awakening score,^[16]^ DR was defined as Steward scores <4 >120 minutes after stoppage of anesthetics. According to the occurrence of DR, the patients included in the study were divided into DR group and control group.

### 2.5 Statistical analysis

All the collected data were input into and processed with SPSS 22.0 statistical software for data analysis. *t* Test and Chi-square test were used to analyze the characteristics and treatment details of patients. Besides, we had included in multivariable logistic regression model including all variables that make sense clinically, even if they were insignificant at univariate analyses, to identify the potential influencing factors for DR in patients with laparoscopic radical biliary surgery.^[17]^*P* < .05 was considered statistically significant in this study, and all of the tests were 2-sided.

## 3 Results

### 3.1 Characteristics of included patients

Overall, 180 patients with laparoscopic radical biliary surgery were identified initially, we excluded 12 patients with regards to incomplete medical record. In total, 168 patients with laparoscopic radical biliary surgery were included finally, of whom 42 patients occurred the DR, the incidence of DR in patients with laparoscopic radical biliary surgery was 25%. As showed in Table 1, there were significant differences regarding the age and BMI between DR group and control group (all *P* < .05), and there were not significant differences regarding sex, smoking, hypertension, diabetes, hyperlipidemia, and anemia between DR group and control group (all *P* > .05).

**Table 1:** Characteristics of included patients.

Variables	DR group (n = 42)	Control group (n = 126)	*t*/*χ*^2^	*P*
Male/female	31/11	90/36	1.203	.094
Age, yr	73.03 ± 9.11	67.35 ± 10.12	10.406	.041
BMI, kg/m^2^	27.45 ± 3.21	24.13 ± 3.44	7.524	.028
Alcohol drinking	28 (66.67%)	89 (68.99%)	1.180	.097
Smoking	15 (35.71%)	52 (41.27%)	1.094	.101
Hypertension	29 (69.05%)	88 (69.84%)	1.182	.055
Diabetes	18 (42.85%)	50 (39.68%)	1.225	.073
Hyperlipidemia	11 (26.19%)	37 (29.37%)	1.136	.072
Anemia	9 (21.43%)	29 (23.02%)	1.085	.086

### 3.2 Treatment details comparison of 2 groups

As presented in Table 2, there were significant differences regarding the duration of surgery, duration of anesthesia, and use of intraoperative combined sevoflurane inhalation (all *P* < .05), and there were no significant differences regarding American Society of Anesthesiologists, New York Heart Association, TNM, and estimated blood loss between DR group and control group (all *P* > .05).

**Table 2:** Comparison of treatment details of 2 groups.

Variables	DR group (n = 42)	Control group (n = 126)	*t*/*χ*^2^	p
ASA				
Level II	35 (83.33%)	99 (78.57%)	1.143	.093
Level III	7 (16.67%)	27 (21.43%)		
NYHA				
Level I	30 (71.43%)	87 (69.05%)	1.104	.088
Level II	10 (23.81%)	31 (24.60%)		
Level III	2 (4.76%)	8 (66.35%)		
TNM				
Level I	12 (28.57%)	39 (30.95%)	1.028	.156
Level II	16 (38.09%)	51 (40.47%)		
Level III	14 (33.33%)	36 (28.57%)		
Duration of surgery, min	238.07 ± 28.25	210.53 ± 32.11	32.063	.031
Duration of anesthesia, min	245.18 ± 21.22	205.12 ± 23.93	31.034	.018
Intraoperative combined sevoflurane inhalation	31 (73.81%)	50 (39.68%)	1.406	.011

### 3.3 Logistic regression analysis on the risk factors for DR

Table 3 demonstrates the assessment of variables of multivariable logistic regression. As Table 4 presented, multivariable logistic regression analysis showed that age ≥70 years (odd ratio [OR] 1.454, 95% confidence interval [CI] 1.146–1.904), BMI ≥25 kg/m^2^ (OR 1.303, 95% CI 1.102–1.912), alcohol drinking (OR 2.041, 95% CI 1.336–3.085), smoking (OR 1.128, 95% CI 1.007–2.261), duration of surgery ≥220 minutes (OR 1.239, 95% CI 1.039–1.735), duration of anesthesia ≥230 minutes (OR 1.223, 95% CI 1.013–1.926), intraoperative combined sevoflurane inhalation (OR 1.207, 95% CI 1.008–1.764) were the independent risk factors for DR in patients with radical biliary surgery (all *P* < .05).

**Table 3:** Assessment of variables of multivariable logistic regression.

Factors	Variables	Assignment
DR	Y	Yes = 1, no = 2
Gender	X_1_	Male = 1, female = 2
Age, yr	X_2_	≥70 = 1, <70 = 2
BMI, kg/m^2^	X_3_	≥25 = 1, <25 = 2
Alcohol drinking	X_4_	Yes = 1, no = 2
Smoking	X_5_	Yes = 1, no = 2
Hypertension	X_6_	Yes = 1, no = 2
Diabetes	X_7_	Yes = 1, no = 2
Hyperlipidemia	X_8_	Yes = 1, no = 2
Anemia	X_9_	Yes = 1, no = 2
ASA	X_10_	Level III = 1, level II = 2
NYHA	X_11_	Level III = 1, level II = 2, level I = 3
TNM	X_12_	Level III = 1, level II = 2, level I = 3
Duration of surgery, min	X_13_	≥220 = 1, <220 = 2
Duration of anesthesia, min	X_14_	≥230 = 1, <230 = 2
Intraoperative combined sevoflurane inhalation	X_15_	Yes = 1, no = 2

**Table 4:** Logistic regression analysis on the risk factors for DR in patients who underwent radical biliary surgery.

Variables	*β*	SE	OR	95% CI	*P*
Age ≥70 yr	0.185	0.202	1.454	1.146∼1.904)	.048
BMI ≥25 kg/m^2^	0.122	0.319	1.303	1.102∼1.912	.037
Alcohol drinking	0.119	0.121	2.041	1.336∼3.085	.042
Smoking	0.176	0.114	1.128	1.007∼2.261	.018
Duration of surgery ≥220 min	0.105	0.103	1.239	1.039∼1.735	.011
Duration of anesthesia ≥230 min	0.173	0.110	1.223	1.013∼1.926	.015
Intraoperative combined sevoflurane inhalation	0.118	0.106	1.207	1.008∼1.764	.043

## 4 Discussions

Previous studies^[18,19]^ indicate that the incidence of DR in patients with laparoscopic radical biliary surgery is high, and it has adverse effects on the short-term and long-term prognosis of patients. In this study, the incidence of DR in patients with laparoscopic radical biliary surgery was 25%, which was consistent with previous reports. Taking into account the potential correlation between DR and related complications, early identification, diagnosis and intervention of high-risk patients for DR have clinically significance to the prognosis of patients.^[20–22]^ The results of this present study indicate that age ≥70 years, BMI ≥25 kg/m^2^, alcohol drinking, smoking, duration of surgery ≥220 minutes, duration of anesthesia ≥230 minutes, and intraoperative combined sevoflurane inhalation were the independent risk factors for DR in patients with radical biliary surgery (all *P* < .05).

The time for patients with general anesthesia to recover from anesthesia mainly depends on the metabolic rate of analgesic drugs.^[23]^ In recent years, the comprehensive evaluation of patients before surgery and the continuous development of anesthetic drugs and monitoring technology have reduced the occurrence of anesthesia DR.^[24]^ The rate gradually declines, and most patients can wake up quickly after surgery, but the phenomenon of delayed wake-up still occurs, which brings a huge challenge to medical staff related to anesthesia.^[25]^ The basic patient factors include age, sex, genetic factors, and preoperative combined underlying diseases.^[26]^ Some authors^[27,28]^ hypothesize that there is a clear relationship between age and the occurrence of DR. The elderly are more likely to experience DR after surgery. The reason may be that the function of the body's central nervous system decreases with age. The clearance rate and plasma protein binding rate of drugs are reduced, and the free plasma concentration of the drug is higher, so the metabolism of anesthetic drugs is slower.

Smoking patients generally have varying degrees of pulmonary ventilatory dysfunction. Smoking can induce small airway remodeling, and the reduction of lung function is the case mechanism leading to hypoxemia.^[29]^ Smoking can also increase HbCO in the blood, causing the dissociation curve of oxidized hemoglobin to shift to the left, and the tighter combination of Hb and oxygen makes it more difficult for tissues to use oxygen, which leads to DR.^[30]^ Drinking alcohol is a risk factor for DR. After ethanol enters liver cells, it is oxidized to acetaldehyde through 2 pathways of liver alcohol dehydrogenase, hydrogen peroxide decomposition enzyme, and liver microsomal alcohol oxidase. A large amount of acetaldehyde has obvious effects on liver cells. Its toxic effects directly and indirectly lead to liver cell degeneration, necrosis and fibrosis, and can develop into cirrhosis or even liver cancer in severe cases.^[31]^ Most anesthetics and analgesics are decomposed by the liver and increase the burden on the liver, which leads to prolonged and enhanced antagonistic effects of anesthetics and muscle relaxants,^[32]^ which easily leads to postoperative DR.

In patients with higher BMI, there are dual factors of increased total dose of anesthetic drugs and slower metabolism. Therefore, the risk of DR is significantly higher than that of patients with normal BMI.^[33]^ However, there are fewer studies on the specific cut-off value of BMI. Some authors^[34–36]^ believe that awakening after anesthesia is usually related to the genetic modifications of the drug receptor or target. For example, the polymorphic changes of the gamma-aminobutyric acid receptor may make the anesthetic drug propofol in the body, and the rate of metabolism slows down accordingly. Studies^[37,38]^ have shown that patients with preoperative heart disease, hypertension, and diabetes are prone to delayed recovery even if they undergo surgery unrelated to the comorbidities, but the specific mechanism is still unclear. In the perioperative factors of DR from anesthesia, the main factors are the dosage of anesthetic drugs, the patient's body temperature and duration of surgery, and the amount of intraoperative fluid infusion.^[39]^ Patients who are lighter or have liver and kidney function abnormalities who are not carefully evaluated before surgery, may be given overdosed anesthetic drugs, which may lead to high drug concentration in the body and DR.^[40]^ If the operation time is very long and the evaluation of the end of the operation is biased, the anesthesia duration will be too long accordingly,^[41]^ which is also an important factor for occurrence of DR from anesthesia. It must be noted that patients with lower body temperature who enter the recovery room after surgery are more likely to develop DR.^[42]^ Drops in body temperature may decrease the activity of enzymes related to drug metabolism, which may cause drugs to accumulate in the body and patients experience DR.^[43]^

Several limitations in this present study must be considered. Firstly, here is a high correlation between the amount of intraoperative fluid infusion and the delay in awakening after anesthesia.^[44,45]^ Larger volumes of intraoperative fluid infusion increases the chance of delayed awakening after surgery.^[46]^ However, this mechanism is unclear. In this present study, due to the lack of related data, we cannot include those factors into further analysis, future studies with larger sample size and more comprehensive factors are needed. Secondly, we chosen the cutoffs because our study was a retrospective design with small sample size, it would be better if we could build a model with time to recovery from anesthesia stop as a continuous variable, duration of surgery and duration of anesthesia used as continuous variables to evaluate the influence on DR, which will provide more reliable evidence into the clinical management of DR. Therefore, prospective studies on this topic are needed in the future.

## 5 Conclusions

In conclusions, we have found that age ≥70 years, BMI ≥25 kg/m^2^, alcohol drinking, smoking, duration of surgery ≥220 minutes, duration of anesthesia ≥230 minutes, and intraoperative combined sevoflurane inhalation were the independent risk factors for DR in patients with radical biliary surgery. Among the relevant factors analyzed in this study, patients’ age and BMI are high-risk factors that cannot be corrected immediately, but operation and anesthesia duration, intraoperative anesthetic drug use are factors that can be improved. On the one hand, clinicians should ensure patient safety and surgery under the premise of curative effect, accurately assess the patient's dosage of anesthetics, and minimize the operation time and the use time of anesthetics; on the other hand, it's necessary to accurately identify high-risk patients with delayed awakening, corresponding nursing strategies should be formulated on the basis of routine care, and the training of nursing staff should be strengthened. For patients with older age, higher BMI, and longer surgery and anesthesia time, it is necessary to closely monitor the patient's vital signs to reduce the risk of DR.

## Author contributions

Guohui Zhang, Yingzi Ling designed research; Guohui Zhang, Bingbing Pan, Dan Tan conducted research; Guohui Zhang analyzed data; Guohui Zhang and Yingzi Ling had primary responsibility for final content. All authors read and approved the final manuscript.

**Conceptualization:** Guohui Zhang, Bingbing Pan, Dan Tan, Yingzi Ling.

**Data curation:** Guohui Zhang, Bingbing Pan.

**Formal analysis:** Guohui Zhang, Dan Tan, Yingzi Ling.

**Investigation:** Bingbing Pan, Dan Tan, Yingzi Ling.

**Resources:** Guohui Zhang.

**Software:** Guohui Zhang, Yingzi Ling.

**Supervision:** Guohui Zhang.

**Visualization:** Yingzi Ling.

**Writing – original draft:** Yingzi Ling.

## References

[1] Mizuno T Ebata T Nagino M. Advanced hilar cholangiocarcinoma: An aggressive surgical approach for the treatment of advanced hilar cholangiocarcinoma: perioperative management, extended procedures, and multidisciplinary approaches. Surg Oncol 2020;33:201–6. 31301935 10.1016/j.suronc.2019.07.002

[2] Zhang Y Dou C Wu W. Total laparoscopic versus open radical resection for hilar cholangiocarcinoma. Surg Endosc 2020;34:4382–7. 31664578 10.1007/s00464-019-07211-0

[3] Bednarsch J Czigany Z Lurje I. Left- versus right-sided hepatectomy with hilar en-bloc resection in perihilar cholangiocarcinoma. HPB (Oxford) 2020;22:437–44. 31383591 10.1016/j.hpb.2019.07.003

[4] Birgin E Rasbach E Reissfelder C Rahbari NN. A systematic review and meta-analysis of caudate lobectomy for treatment of hilar cholangiocarcinoma. Eur J Surg Oncol 2020;46:747–53. 31987703 10.1016/j.ejso.2020.01.023

[5] Sapisochin G Ivanics T Subramanian V Doyle M Heimbach JK Hong JC. Multidisciplinary treatment for hilar and intrahepatic cholangiocarcinoma: A review of the general principles. Int J Surg 2020;82S:77–81. 32380231 10.1016/j.ijsu.2020.04.067

[6] Li J Zhou MH Ma WJ Li FY Deng YL. Extended lymphadenectomy in hilar cholangiocarcinoma: what it will bring? World J Gastroenterol 2020;26:3318–25. 32655260 10.3748/wjg.v26.i24.3318 PMC7327786

[7] Trepanier M Valin-Thorburn A Kouyoumdjian A. Intracorporeal versus extracorporeal anastomosis for right colectomy does not affect gastrointestinal recovery within an enhanced recovery after surgery program. Surg Endosc 2020;34:4601–8. 31646437 10.1007/s00464-019-07204-z

[8] Ohta J Suto T Kato D Hiroki T Obata H Saito S. Loss of endogenous analgesia leads to delayed recovery from incisional pain in a rat model of chronic neuropathic pain. Brain Res 2020;1727:11–7. 10.1016/j.brainres.2019.146568 31785233

[9] Zhang XY Zhang XZ Lu FY. Factors associated with failure of enhanced recovery after surgery program in patients undergoing pancreaticoduodenectomy. Hepatobiliary Pancreat Dis Int 2020;19:51–7. 31563597 10.1016/j.hbpd.2019.09.006

[10] Zhu J Li X Li H. Enhanced recovery after surgery pathways benefit patients with soft pancreatic texture following pancreaticoduodenectomy. Am J Surg 2020;219:1019–23. 31409454 10.1016/j.amjsurg.2019.08.002

[11] Mansour JC Aloia TA Crane CH Heimbach JK Nagino M Vauthey JN. Hilar cholangiocarcinoma: expert consensus statement. HPB (Oxford) 2015;17:691–9. 26172136 10.1111/hpb.12450 PMC4527854

[12] Li JD Zhao ZL. [Current status of laparoscopic techniques in the surgical treatment of biliary carcinoma]. Zhonghua Wai Ke Za Zhi 2018;56:338–41. 29779308 10.3760/cma.j.issn.0529-5815.2018.05.004

[13] Yao H Xiu D Fu W. [Outcomes evaluation of laparoscopic radical coloproctectomy and hepatectomy for resectable colorectal cancer with liver metastases]. Zhonghua Wai Ke Za Zhi 2014;52:919–23. 25622583

[14] Li Y Li XQ Kang Y Liu J. [Determination of dosage and effectiveness of propofol and ketamine for TIVA in adults]. Sichuan Da Xue Xue Bao Yi Xue Ban 2014;45:451–6. 24941816

[15] Yildiz M Tavlan A Tuncer S Reisli R Yosunkaya A Otelcioglu S. Effect of dexmedetomidine on haemodynamic responses to laryngoscopy and intubation: perioperative haemodynamics and anaesthetic requirements. Drugs R D 2006;7:43–52. 16620136 10.2165/00126839-200607010-00004

[16] Aldrete JA Kroulik D. A postanesthetic recovery score. Anesth Analg 1970;49:924–34. 5534693

[17] Lederer DJ Bell SC Branson RD. Control of confounding and reporting of results in causal inference studies. guidance for authors from editors of respiratory, sleep, and critical care journals. Ann Am Thorac Soc 2019;16:22–8. 30230362 10.1513/AnnalsATS.201808-564PS

[18] Perinel J Mariette C Dousset B. Early enteral versus total parenteral nutrition in patients undergoing pancreaticoduodenectomy: a randomized multicenter controlled trial (Nutri-DPC). Ann Surg 2016;264:731–7. 27429039 10.1097/SLA.0000000000001896

[19] Cao D Li X Zhang H. Factors influencing the long-term prognosis of hilar cholangiocarcinoma. J Capital Univ Med Sci 2020;41:125–9.

[20] Takamoto T Hashimoto T Inoue K. Applicability of enhanced recovery program for advanced liver surgery. World J Surg 2014;38:2676–82. 24838485 10.1007/s00268-014-2613-0

[21] Warner S McKiernan PJ Hartley J. Hepatopulmonary syndrome in children: a 20-year review of presenting symptoms, clinical progression, and transplant outcome. Liver Transpl 2018;24:1271–9. 30066494 10.1002/lt.25296

[22] di Sebastiano P Festa L De Bonis A. A modified fast-track program for pancreatic surgery: a prospective single-center experience. Langenbecks Arch Surg 2011;396:345–51. 20703500 10.1007/s00423-010-0707-1

[23] Wong-Lun-Hing EM van Dam RM van Breukelen GJ. Randomized clinical trial of open versus laparoscopic left lateral hepatic sectionectomy within an enhanced recovery after surgery programme (ORANGE II study). Br J Surg 2017;104:525–35. 28138958 10.1002/bjs.10438

[24] Choi SS Cho SS Ha TY Hwang S Lee SG Kim YK. Intraoperative factors associated with delayed recovery of liver function after hepatectomy: analysis of 1969 living donors. Acta Anaesthesiol Scand 2016;60:193–202. 26830214 10.1111/aas.12630

[25] Huang H Deng M Jin H Liu A Dahmen U Dirsch O. Reduced hepatic arterial perfusion impairs the recovery from focal hepatic venous outflow obstruction in liver-resected rats. Transplantation 2014;97:1009–18. 24770620 10.1097/TP.0000000000000089

[26] Dichtwald S Ben-Haim M Papismedov L Hazan S Cattan A Matot I. Intrathecal morphine versus intravenous opioid administration to impact postoperative analgesia in hepato-pancreatic surgery: a randomized controlled trial. J Anesth 2017;31:237–45. 27885425 10.1007/s00540-016-2286-y

[27] Kim D Shin BS Song I. Relationship between intraoperative bispectral index and consciousness recovery in patients with hepatic encephalopathy undergoing liver transplant: a retrospective analysis. Transplant Proc 2019;51:798–804. 30979467 10.1016/j.transproceed.2018.10.031

[28] Marsman HA de Graaf W Heger M. Hepatic regeneration and functional recovery following partial liver resection in an experimental model of hepatic steatosis treated with omega-3 fatty acids. Br J Surg 2013;100:674–83. 23456631 10.1002/bjs.9059

[29] Andersson M Blanc PD Toren K Jarvholm B. Smoking, occupational exposures, and idiopathic pulmonary fibrosis among Swedish construction workers. Am J Ind Med 2021;64:251–7. 33547652 10.1002/ajim.23231

[30] Bellou V Belbasis L Evangelou E. Tobacco smoking and risk for pulmonary fibrosis: a prospective cohort study in UK Biobank. Chest 2021;16:12–20. 10.1016/j.chest.2021.04.035 33905677

[31] Phan THG Paliogiannis P Nasrallah GK. Emerging cellular and molecular determinants of idiopathic pulmonary fibrosis. Cell Mol Life Sci 2021;78:2031–57. 33201251 10.1007/s00018-020-03693-7 PMC7669490

[32] Vassar RL Rose J. Motor systems and postural instability. Handb Clin Neurol 2014;125:237–51. 25307579 10.1016/B978-0-444-62619-6.00015-X

[33] Fukazawa K Yamada Y Gologorsky E Arheart KL Pretto EA Jr. Hemodynamic recovery following postreperfusion syndrome in liver transplantation. J Cardiothorac Vasc Anesth 2014;28:994–1002. 25107717 10.1053/j.jvca.2014.02.017

[34] Lillemoe HA Marcus RK Kim BJ Narula N Davis CH Aloia TA. Detours on the road to recovery: what factors delay readiness to return to intended oncologic therapy (RIOT) after liver resection for malignancy? J Gastrointest Surg 2019;23:2362–71. 30809785 10.1007/s11605-019-04165-5 PMC6900935

[35] Yang HR Thorat A Jeng LB. Living donor liver transplantation in acute liver failure patients with grade iv encephalopathy: is deep hepatic coma still an absolute contraindication? A successful single-center experience. Ann Transplant 2018;23:176–81. 29531210 10.12659/AOT.907274 PMC6248027

[36] Suh SJ Yim HJ Yoon EL. Is propofol safe when administered to cirrhotic patients during sedative endoscopy? Korean J Intern Med 2014;29:57–65. 24574834 10.3904/kjim.2014.29.1.57 PMC3932396

[37] Matsuo K Rocha FG Ito K. The Blumgart preoperative staging system for hilar cholangiocarcinoma: analysis of resectability and outcomes in 380 patients. J Am Coll Surg 2012;215:343–55. 22749003 10.1016/j.jamcollsurg.2012.05.025

[38] de Jong MC Marques H Clary BM. The impact of portal vein resection on outcomes for hilar cholangiocarcinoma: a multi-institutional analysis of 305 cases. Cancer 2012;118:4737–47. 22415526 10.1002/cncr.27492

[39] Ito F Agni R Rettammel RJ. Resection of hilar cholangiocarcinoma: concomitant liver resection decreases hepatic recurrence. Ann Surg 2008;248:273–9. 18650638 10.1097/SLA.0b013e31817f2bfd

[40] Dilek ON Gungor F Acar T. The role of portoenterostomy with aggressive hilar dissection in biliary tract tumors: report of case series and review of the literature. Indian J Surg 2020;11:01–7. 10.1007/s12262-020-02259-y PMC7222060 32410790

[41] Wang G Wang Q Fan X Ding L Dong L. The significance of adjuvant therapy for extrahepatic cholangiocarcinoma after surgery. Cancer Manag Res 2019;11:10871–82. 31920396 10.2147/CMAR.S224583 PMC6941596

[42] Hu HJ Jin YW Shrestha A. Predictive factors of early recurrence after R0 resection of hilar cholangiocarcinoma: a single institution experience in China. Cancer Med 2019;8:1567–75. 30868740 10.1002/cam4.2052 PMC6488134

[43] Furuta T Yamaguchi M Nakagami R. Delayed hepatic signal recovery on ferucarbotran-enhanced magnetic resonance images: an experimental study in rat livers with gadolinium chloride-induced Kupffer cell damage. MAGMA 2013;26:313–24. 23117343 10.1007/s10334-012-0354-3

[44] Seo H Jun IG Ha TY Hwang S Lee SG Kim YK. High stroke volume variation method by mannitol administration can decrease blood loss during donor hepatectomy. Medicine (Baltimore) 2016;95:24–8. 10.1097/MD.0000000000002328 PMC4718235 26765409

[45] Choi SS Jun IG Cho SS Kim SK Hwang GS Kim YK. Effect of stroke volume variation-directed fluid management on blood loss during living-donor right hepatectomy: a randomised controlled study. Anaesthesia 2015;70:1250–8. 26215206 10.1111/anae.13155

[46] Choi SS Kim SH Kim YK. Fluid management in living donor hepatectomy: recent issues and perspectives. World J Gastroenterol 2015;21:12757–66. 26668500 10.3748/wjg.v21.i45.12757 PMC4671031

